# Adherence to National Asthma Guidelines in the Acute Care Setting: A Retrospective Cohort Study

**DOI:** 10.7759/cureus.48725

**Published:** 2023-11-13

**Authors:** Muhammad Hussain, Taimoor Hassan, Gulmeena Hussain, Muhammad Zahir Shah, Zoe Kimbley

**Affiliations:** 1 Medicine, University Hospitals Birmingham NHS Foundation Trust, Birmingham, GBR; 2 Medicine, Bassetlaw Hospital, Worksop, GBR; 3 Gastroenterology, University Hospitals Birmingham NHS Foundation Trust, Birmingham, GBR; 4 Acute Medicine, University Hospitals Birmingham NHS Foundation Trust, Birmingham, GBR

**Keywords:** airways disease, scottish intercollegiate guidelines network (sign), asthma severity, british thoracic society (bts), peak expiratory flow rate (pefr)

## Abstract

Background: Peak flow is a crucial but simple test used to categorize the severity of an episode of an acute exacerbation of asthma. It should be regularly done in all the patients who present with asthma acute exacerbation in the emergency department. The British Thoracic Society (BTS)/Scottish Intercollegiate Guidelines Network (SIGN) guidelines stipulate peak flow use as one of the main tools to categorize acute asthma into moderate, severe, and life-threatening asthma. The BTS and SIGN guidelines also state peak flow is to be utilized in monitoring the disease and to guide in treating patients with acute asthma.

Methods and materials: This study aims to identify the adherence to BTS/SIGN guidelines around the use of peak expiratory flow rate (PEFR) in assessing the severity of patients presenting with acute exacerbation of asthma in a district general hospital. The retrospective cohort study involved collating data between October 2022 and February 2023 from our hospital electronic system. The data collected about the use of PEFR and whether the patients were being classified by severity in presentation following this was compared to the BTS/SIGN 158 asthma guidelines. Following this, the data analysis was done using IBM SPSS Statistics for Windows, Version 21.0 (Released 2012; IBM Corp., Armonk, New York, United States).

Results: Data from 92 patients were collated. PEFR was recorded for 29.3% (n=27) of patients and acute exacerbation of asthma severity was documented in merely 17.4% (n=16) patients.

Conclusion: The results indicate a significant proportion of the patient cohort analyzed did not have peak flow readings, there is clear room for improvement, and further intervention is needed in order for the department to adhere to the gold standard guidelines (i.e., BTS/SIGN 158), and thus improve the management and monitoring of acute asthma exacerbations. Future directions can include departmental education, posters as a reminder, and prompts on the electronic system used to alert users to check PEFR when a diagnosis of acute asthma exacerbation is documented.

## Introduction

Approximately 262 million people globally suffered from asthma in 2019, with the figures increasing annually, according to the World Health Organization (WHO), which states this association is due to increased urbanization [1]. Asthma affects all age groups with significant morbidity. A prompt diagnosis and reasonable management plan decrease morbidity. It also decreases healthcare costs and side effects caused by treatment [2].

Asthma is a chronic, obstructive airway disease, defined by the presence of more than one symptom of shortness of breath, chest tightness, wheezing and cough associated with variable airflow obstruction according to the British Thoracic Society (BTS) and Scottish Intercollegiate Guidelines Network (SIGN). It can also be defined as airway hyperresponsiveness and airway inflammation as components of the disease [3]. The National Institute of Clinical Effectiveness (NICE) propose that an asthma diagnosis should not be made if there is no evidence of airway obstruction or inflammation [4].

The pathophysiology of acute exacerbation of asthma and its clinical signs includes the release of inflammatory mediators, mast cells, lymphocytes, and macrophages. These symptoms lead to airway obstruction, chest tightness, and coughing, particularly at night or early morning [5]. Hyperinflation of the lungs is seen in the chest x-ray of acute asthma [6].

BTS/SIGN guidelines are the current gold standard to be adhered to within the United Kingdom (UK). Peak expiratory flow rate (PEFR) is used as an initial tool. Peak expiratory flow (PEF) is used in categorising the severity of asthma; 50-70% is labelled as moderate asthma, PEF of 33-50% as acute severe asthma, and PEF <33% is termed as life-threatening asthma.

This study has been designed to explore how effectively gold-standard protocol for acute asthma exacerbation management, and assortment of the episode’s severity has been implemented in the emergency department and acute medical unit at a district general hospital in Birmingham.

This article has been accepted as a Poster Presentation at the Acute and General Medical Conference, London, and will be presented on November 14, 2023.

## Materials and methods

This is a retrospective cohort study carried out in accordance with the Declaration of Helsinki. It is a study of asthma management according to the BTS/SIGN guidelines carried out at a district general hospital, Good Hope Hospital, in Birmingham, UK. The primary outcome measure was how well the severity of the asthma exacerbation and peak flow had been documented in both the ED and the acute medical unit (AMU). Secondary outcome measures included whether the patients went to the intensive therapy unit (ITU), and were seen by the respiratory registrar or speciality nurse.

The patients included in the study had their acute asthma exacerbation treated at the hospital between October 2022 and February 2023. This study included only adults (aged over 17). For each of these patients, a questionnaire tool was created against the BTS/SIGN guidelines to document how closely the treatment of the patients adhered to the gold standard guidelines. Details collected included: patient ID, age, ethnicity, date of presentation to the department, whether peak flow was recorded, and whether the classification of the severity of the acute asthma exacerbation was recorded (in accordance with BTS/SIGN guidelines). This was collated onto an Excel spreadsheet (Microsoft Corporation, Redmond, Washington, United States), to clearly display the results and the data were then evaluated descriptively as percentages.

Patients eligible for inclusion were identified via the hospital’s local information technology (IT)/data team. The data for each of these patients on the electronic patient records system was then analyzed in further detail using IBM SPSS Statistics for Windows, Version 21.0 (Released 2012; IBM Corp., Armonk, New York, United States).

## Results

In our study, 92 patients were selected, aged 17-96 years old. Sixty-seven patients were female and 25 were male. A summary of the patient demographics can be seen in Table 1.

**Table 1:** Demographics of the patients included in the study.

Demographics		% (n)
Sex	Male	27.2 (25)
	Female	72.8 (67)
Age Range		79

The data was compared against BTS/SIGN guidelines to observe the percentage of patients managed using PEFR and to document the severity of the patients. It was observed that in the ED, severity was documented in only 16 patients while in the AMU, the severity was documented in only eight patients. PEFR was documented in the ED in about 27 patients while in the AMU, in about 39 patients (Table 2). Out of the total cohort, only 2.2% (n=2) were admitted to the ICU, and 58.7% (n=54) were reviewed by a respiratory registrar or specialty nurse.

**Table 2:** Comparison of findings with BTS/SIGN guidelines AMU: acute medical unit; ITU: intensive therapy unit

Criteria from the guidelines		Yes, % (n)	No, % (n)
Severity documented	ED	17.4 (16)	82.6 (76)
	AMU	8.7 (8)	91.3 (84)
Peak flow documented	ED	29.3 (27)	70.7 (65)
	AMU	42.4 (39)	57.6 (53)
ITU Admission		2.2(2)	97.8 (90)
Respiratory registrar/specialty nurse review		58.7(54)	41.3(38)

**Figure 1:**
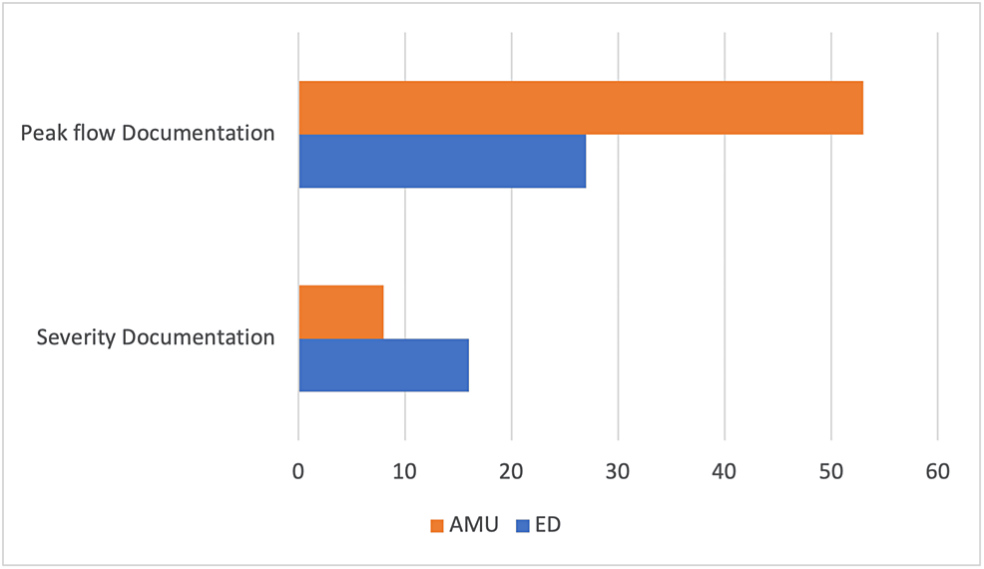
Proportions of patients who had documentation of peak flow and severity of asthma in the ED and AMU AMU: acute medical unit

The recording of severity of the asthma exacerbation was better in ED as compared to that of AMU. Contrary to that, recording of peak flow was more consistent in AMU (Figure 1).

## Discussion

Asthma is one of the most common non-contagious ailments. Among the causes of years lived with disability, asthma ranks 16th around the globe [7]. The most common form is childhood-onset asthma, though some patients can develop it later in life; this is known as late-onset asthma and is typically more severe and less associated with allergies as compared to childhood-onset asthma [8]. An estimated 10% of asthma-related admissions get admitted to the ITU [9]. The mortality rate is high in asthma because of the failure to identify its risk factors [10].

PEF or forced expiratory volume (FEV1) measures the calibre of the airway. In an acute setting, PEF is more suitable. PEF is a “percentage of predicted” and it is more useful when measured and put into the context of information regarding the patient’s own best PEF reading. The best reading of the three is taken as the one to be documented. Where this information is not available, it still provides an approximation that can aid and guide management and classification. The same type of peak flow meter has to be used to ensure the readings taken are accurate and to reduce external factors that could alter them [11]. The Global Initiative for Asthma (GINA) guidelines allude to the use of PEFR monitoring to assess response to treatment, to identify any triggers for worsening symptoms, and provide an initial baseline value to guide the management plan [12]. 

Treatment of acute severe asthma includes oxygen supplementation of 94-98% oxygen-driven beta-2-agonist bronchodilators to relieve bronchospasms and, most importantly, prednisolone for at least five days. Ipratropium bromide can be added, which can reverse bronchospasm faster [9].

The SIGN158 published in July 2019 divides asthma into four categories based on severity: moderate, acute severe, life-threatening asthma, and near-fatal asthma; this is concluded based on PEF and other clinical findings. PEF of 50-75% or best predicted, and oxygen saturation (SpO2) of more than 92% is labelled as moderate asthma. Likewise, a PEF of <50% best or predicted is labelled as acute severe asthma. Other features include respiratory rate >25%/min, SpO2 of >92%, pulse >110, and inability to finish a sentence in one breath. A PEF of <33%, hypotension, silent chest, cyanosis, arrhythmia, altered consciousness, poor respiratory effort, hypotension, and exhaustion indicate life-threatening asthma. Mechanical ventilation requirement with increased inflation pressure or high partial pressure of CO2 is categorized as near-fatal asthma. [11]

In a study by Protheroe et al., it was seen that PEFR was recorded in 63.0% of patients [13]. In another study, 293 patients were seen for a presentation of acute asthma, of whom the severity of their asthma was assessed in only 31% [14]. PEFR was recorded in 62% of the patients. In another clinical audit by Bayes et al., the classification of asthma severity was documented in 55% in the first cycle and 66% in the second cycle [15]. Of this, the severity of 33% in the first cycle and 21% in the second cycle was assessed wrong, and all life-threatening attacks were not identified. 

A study done in 2013 in Saudi Arabia on the Saudi National Asthma Protocol (SNAP) showed that the main factor of non-compliance to the guidelines is due to a lack of awareness of the protocol among primary care doctors and paediatricians. [16]

The limitation of our study was that it was a single-centre study and patients were selected from electronic patient records and those who presented with an upper respiratory infection and asthma might have been missed. In our study, it was observed that in the ED, severity was documented in 16% of the patients, and peak flow was documented in approximately 27% of the patients. The reason behind this low percentage could be that the early categorisation of patients with asthma exacerbation in the ED helps ED doctors to initiate the most appropriate immediate management of the patient. Secondly, the low percentage could be due to a lack of prompts on the post-intensive care syndrome (PICS) for documenting asthma severity. Since our peak flow and severity documentation were very poor, there must be regular audits, and teaching among doctor colleagues to keep them up-to-date regarding the updated guidelines of acute asthma exacerbation.

## Conclusions

This study compared the data recorded in the electronic patient system in a single hospital to the BTS/SIGN guidelines and found a substantial proportion of patients’ severity of asthma was not properly documented and peak flow readings were not taken for a great proportion of the patients either as part of their assessment.

To improve these shortcomings, interventions are needed such as departmental education or presentations, prompts on the electronic system, and posters to remind doctors and nurses in the ED to take peak flow readings and classify the severity of the exacerbations, in keeping with the gold standard guidelines. This, in turn, will better guide the management of asthma exacerbations and improve patient outcomes.
